# “SQiD, the Single Question in Delirium; can a single question help clinicians to detect delirium in hospitalised cancer patients?” running heading Single Question in Delirium” (Bcan-D-20-01665)

**DOI:** 10.1186/s12885-020-07504-x

**Published:** 2021-01-18

**Authors:** Megan B. Sands, Swapnil Sharma, Lindsay Carpenter, Andrew Hartshorn, Jessica T. Lee, Sanja Lujic, Megan E. Congdon, Angus M. Buchanan, Meera Agar, Janette L. Vardy

**Affiliations:** 1 University of New South Wales, Prince of Wales Clinical School, Sydney, NSW Australia; 2 Department of Palliative and Supportive Care, Nelune Comprehensive Cancer Centre, Prince of Wales Hospital and Community Health Service, Randwick, NSW 2031 Australia; 3 Psychological Medicine Centre, Oxford University Hospitals NHS Foundation Trust, Oxford, UK; 4 Concord Cancer Centre, Concord Repatriation General Hospital, Sydney, NSW Australia; 5 Centre for Big Data Research in Health, University of New South Wales, Sydney, NSW Australia; 6 Gosford Hospital, Gosford, NSW Australia; 7 Centre of Cardiovascular and Chronic Care, University of Technology Sydney Faculty of Health, Ultimo, Australia; 8 Sydney Medical School, University of Sydney, Sydney, NSW Australia

**Keywords:** Delirium, Detection, Screening, Oncology, Cancer, Hospital

## Abstract

**Aim:**

A serious syndrome for cancer in-patients, delirium risk increases with age and medical acuity. Screening tools exist but detection is frequently delayed or missed. We test the ‘Single Question in Delirium’ (SQiD), in comparison to psychiatrist clinical interview.

**Methods:**

Inpatients in two comprehensive cancer centres were prospectively screened. Clinical staff asked informants to respond to the SQiD: “Do you feel that [patient’s name] has been more confused lately?”. The primary endpoint was negative predictive value (NPV) of the SQiD versus psychiatrist diagnosis (Diagnostic and Statistics Manual criteria). Secondary endpoints included: NPV of the Confusion Assessment Method (CAM), sensitivity, specificity and Cohen’s Kappa coefficient.

**Results:**

Between May 2012 and July 2015, the SQiD plus CAM was applied to 122 patients; 73 had the SQiD and psychiatrist interview. Median age was 65 yrs. (interquartile range 54–74), 46% were female; median length of hospital stay was 12 days (5–18 days). Major cancer types were lung (19%), gastric or other upper GI (15%) and breast (14%). 70% of participants had stage 4 cancer. Diagnostic values were similar between the SQiD (NPV = 74, 95% CI 67–81; kappa = 0.32) and CAM (NPV = 72, 95% CI 67–77, kappa = 0.32), compared with psychiatrist interview. Overall the CAM identified only a small number of delirious cases but all were true positives. The specificity of the SQiD was 87% (74–95) The SQiD had higher sensitivity than CAM (44% [95% CI 41–80] vs 26% [10–48]).

**Conclusion:**

The SQiD, administered by bedside clinical staff, was feasible and its psychometric properties are now better understood. The SQiD can contribute to delirium detection and clinical care for hospitalised cancer patients.

**Supplementary information:**

**Supplementary information** accompanies this paper at 10.1186/s12885-020-07504-x.

## Key message

The clinical diagnosis of delirium is often missed, leading to poor outcomes for cancer inpatients such as increased morbidity, mortality, distress and length of stay. Improved delirium detection is an important component of comprehensive cancer care. Compared with a psychiatrist interview, the SQiD - a Single Question in Delirium- showed good negative predictive value [NPV 75% (95%CI 67–81)] and fair agreement [kappa = 0.32 (95%CI 0.08–0.56)]. Specificity of the SQiD was 86% and sensitivity was 44%. The short Confusion Assessment Method (CAM) is a very commonly used delirium detection tool, in our setting the CAM had good specificity (100%) but a sensitivity of 26% and NPV of 72% (95%CI 67–77). The SQiD can contribute to quality of care for cancer patients.

## Background

Delirium is a syndrome defined in terms of neuro-cognitive features and level of awareness, with inattention a salient feature [1]. It is common and associated with poor outcomes for patients, staff and carers. Delirium disproportionately affects medically unwell people, is often reversible but diagnosis is frequently missed. To improve detection, screening for delirium has been advocated in hospitalised patients [2]. An ideal tool for detecting delirium would have good sensitivity, specificity, and negative predictive value (NPV) for all sub-types of delirium [3], be quick to administer and have realistic training requirements. It would be validated against an appropriate research standard [3].

Delirium detection tools for hospitalised patients have been reviewed elsewhere [4, 5], however, with respect to clinical uptake, particularly outside areas where delirium may be considered core business such as specialist aged care or perioperative settings, uptake is largely unknown. Regardless of setting, the use of detection tools in hospital wards may be constrained by several issues such as for example, the need for specific training, added complexity in administration of composite measures and the consideration that all tools take at least a few minutes to administer.

The uptake of routine delirium screening in oncology in-patient settings is not known; only a few detection tools have been tested in oncology in-patients; mostly in “stand-alone” palliative care in-patient units or subsets of oncology patients such as those referred to palliative care or psychiatry services. At the time of study design, three tools, the Mini Mental State Examination (MMSE), Memorial Delirium Assessment Scale (the MDAS, a delirium severity assessment tool) and the Confusion Assessment Method (CAM) [6], were most commonly reported in delirium detection studies in patients with cancer. Subsequently, the Nursing Delirium Rating Scale (Nu-DESC) [7] and “4 A’s test” (4AT) [8] have been favourably tested in “stand alone” palliative care settings and Neefjes et al. have compared the Delirium Observation Screening Scale (DOS) to another delirium rating tool the Delirium Rating Scale R 98 [9].

All delirium tools have some methodological issues. The CAM, which has a short version, is the most widely used delirium detection tool in clinical and research settings. When applied by staff well trained in its administration, the CAM achieves overall sensitivity of 94% (95% confidence interval [CI] 91–97%), and specificity of 89% (95% CI 85–94%) [10]. However, the CAM is less simple to use and relies heavily on the skill and training of the operator [6], making it impractical in some clinical settings, especially those with high staff turnover. In addition, even the short CAM must be accompanied by a test of cognition such as the mini-cog [10, 11], which although relatively brief to administer, adds to the total time and complexity of using it as a screening tool [12]. More recent studies have failed to reproduce in clinical research settings the excellent psychometric properties of the CAM when applied in research settings [13, 14].

Feasibility testing for the SQiD, a single-question, informant-response tool for detecting delirium against an established reference standard has previously occurred [15]. The Single Question, study setting and reference standard are unchanged from the feasibility study.

During the study period, there was a change in the DSM, from DSM IV R to DSM 5 and throughout the study the contemporaneous edition of the DSM interview was used, that is either DSM IV R or DSM 5. Of note, disturbed level of consciousness was removed as part of the diagnostic criteria for delirium in the change to DSM 5.

With the overall purpose of decreasing the distress, morbidity and mortality of delirium by improving delirium detection in hospitalised cancer patients, we designed a novel single question tool for delirium detection (the SQiD) and compared it to a robust standard (DSM interview by consultant psychiatrist).

## Methods

### Study design and setting

This cross-sectional, observational study was undertaken as part of a higher research degree by the first author [16]. Patients were recruited from in-patient oncology wards of two comprehensive cancer centres in Sydney, Australia. Existing bedside clinicians undertook training for administration of the CAM. The SQiD was administered as part of usual care carried out by ward clinical staff. There was no specific training for the SQiD; staff were handed a prompt (see supplementary data) and instructed that any conversation that followed should be as they would normally proceed when questioned by patients or family [15]. The primary objective was to determine the negative predictive value (NPV) of the SQiD versus psychiatrist DSM interview. The secondary objectives were to determine: the NPV of the CAM versus the interview and comparison of the NPV of the SQiD and CAM in relation to interview.

### Human research and ethics approval, participant recruitment, consent and study withdrawal

Institutional ethics approval was obtained from the South-Eastern Sydney Local Health District Human Research Ethics Committee (approval number LNR 11/156). Written consent was obtained from each participant or their proxy if they were unable to give informed consent.

**Fig. 1:**
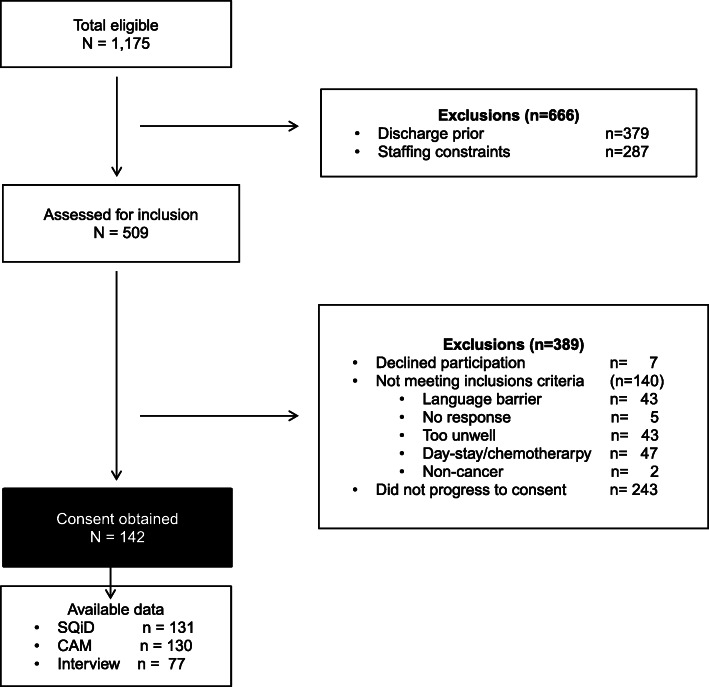
Recruitment Flow diagram.*Interview* Psychiatric Diagnostic and Statistic Manual Interview; *SQiD* Single Question in Delirium; *CAM* Short Confusion Assessment Method

Included patients were adults on a dedicated oncology ward of participating cancer centres, both were within university affiliated acute hospitals. Eligible patients were included regardless of cancer primary site, or stage of disease. Exclusion criteria are listed in Fig. 1. In order to mitigate selection bias, consecutive admissions on nominated recruitment days were targeted for inclusion. At site two, recruitment occurred only on a priori nominated days (Monday to Wednesday) due to staff availability.

Administration of the novel tool (SQiD) and administration of the short CAM (hereafter referred to as CAM) were accepted as consistent with usual care by the ethical review board. Written consent for psychiatrist interview and study participation was specifically sought from the patient or their proxy prior to approach by the psychiatrist but consent occurred after SQiD and CAM administration. This was an intentional part of the study design in order to mitigate training bias via the educational nature of the consent or leading nature of psychiatric interview should it be administered prior to the single question [17].

If the patient was unable to give informed consent, a substitute decision maker was approached. In most instances the SQiD informant was the person (relative, friend or carer) most practically available who could provide history. This was designed to mimic psychiatric co-lateral history but also to be consistent with pragmatic concerns of the clinical staff. In the study, clinical staff were asked to use the person most commonly at the bedside, who they might usually approach with pragmatic questions about the patient. The patient and informants were able to withdraw from the study at any time and were offered the option of consenting for data collected up to withdrawal and where consent for this was granted, data from these subjects were included for analyses. All aspects of the final methodology including use of the SQiD and CAM data from patients who consented to data use but not psychiatrist interview were accepted by the ethical review board.

### Sequence and administration of delirium detection tools and reference standard used for case ascertainment

**Fig. 2:**
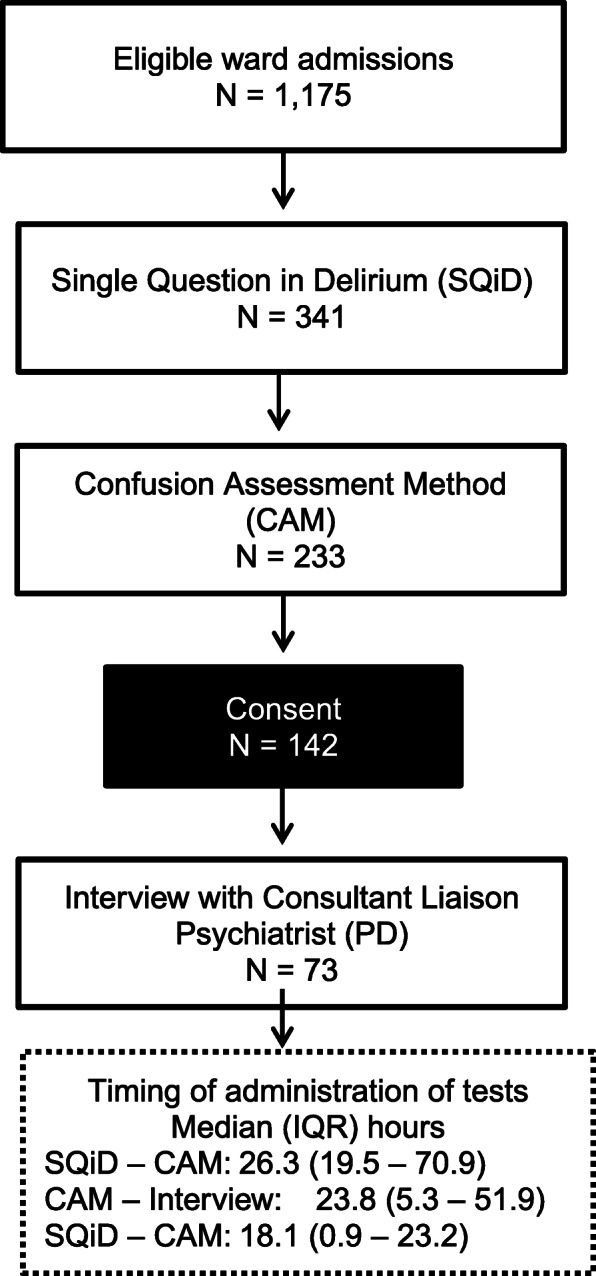
Order of administration of tests, consent and diagnostic reference standard. *Interview* Psychiatric Diagnosic and Statistic Manual Diagnostic Interview; *SQiD* Single Question in Delirium; *CAM* Short Confusion Assessment Method; ; *IQR* Interquartile range

Figure 2 shows the order of administration of tools, consent and reference standard. Study flow was designed to mitigate training or exposure bias of the informant, and for this reason administration of the SQiD always preceded the CAM and the psychiatrist interview was performed last. The CAM, SQiD and psychiatrist interview were all performed by a different assessor, with each blinded to the results of other tests. The psychiatrists had access to the patient’s medical record.

### Administration of the SQiD

A one-hour, in-service education session on delirium was delivered by the investigators at each site (MBS and JL). There was no specific training for the SQiD. Ward clinicians were given a written prompt (see supplementary data) with the SQiD phrase and instructed to administer this verbally and in-person to the most appropriate relative or friend. The SQiD question was not administered as a written questionnaire.

The SQiD was framed: *“Do you feel that [patient’s name] has been more confused lately?”* An interview guide or prompt was used to record the SQiD informant’s yes or no answer to the Single Question together with some demographic information. The text is intended as a prompt for staff administering the SQiD and was not intended to be handed to the SQiD informant. Although only a dichotomous response of “yes” or “no” was recorded by the person administering the SQiD, discussion leading to the decision of which answer was appropriate was not discouraged. This was intended to reflect the real clinical interaction between staff and family/carers a question may encourage. Patients were not specifically included or excluded from this discussion. The SQiD was preferentially administered in person by bedside clinical staff but, if necessary, could be administered over the phone, and was at times administered by a (supervised) health care student as they form part of the usual clinical care team.

### Confusion assessment method (CAM) training and administration

The Short CAM [18] was the CAM version utilised throughout this study. The CAM was administered by senior palliative care and oncology nurses (with 3–10 years of experience on the palliative care consultation team) who received training from a senior aged care nurse familiar with the CAM in daily clinical practice in the aged care assessment team. Study staff administering the CAM were also provided with the Short CAM training manual instructions and tasked with following them [18]. As we wished to balance the rigorous specifications of CAM user training with the real world constrains of clinical staff on the oncology ward, formal inter-rater reliability was not tested. The Mini-Cog [11] was used as the short cognitive test pre-requisite to CAM administration.

### Participant interview by consultant liaison psychiatrist

A post-fellowship consultant liaison psychiatrist (specialist in psychosomatic medicine) assessed each patient in a brief interview, according to the the contemporaneous Diagnostic and Statistical Manual of Mental Disorders (DSM) [19, 20]; classification included delirium motoric subtype. Our aim was for the psychiatric interview to be performed within approximately 24 h of the SQiD and CAM. The psychiatrist had access to the clinical record and could assess fluctuation of symptoms as required for the DSM diagnoses and was also tasked with recording motoric subtype of delirium [21].

### Statistical analysis

Descriptive information is presented in tabular format. Categorical data are presented as total numbers and proportions, and continuous data as medians and interquartile ranges. The difference between groups was tested using Chi-squares and Mann-Whitney tests. Criterion validity between SQiD and CAM tests was measured by estimating diagnostic values of sensitivities, specificities, positive predictive and negative predictive values using the DSM diagnosis as the diagnostic reference standard. Sensitivity was defined as proportion of patients with delirium who were correctly identified using a test [22]*.* Specificity was defined as the proportion of patients without delirium who were correctly identified using a test [22]*.* Cohen’s kappa statistic (kappa) was used to determine the proportion agreement corrected for chance [23]*.* Kappa values above 0.75 denote excellent agreement, 0.40 to 0.75 fair to good agreement, 0.21 to 0.4 as fair and below 0.20 poor agreement. Data management and analysis were undertaken using SAS software, version 9.3 (SAS Institute, Cary NC).

A maximum sample size of 133 was calculated to detect 50% sensitivity (“worst case” scenario) with 20% precision, with an alpha of 5% and an assumed delirium prevalence of 18% [24]. A sample size of 194 was needed to achieve 90% sensitivity with 5% precision.

In instances where there was missing data, participants were only included if they had two complete assessments where data could be used for the respective comparisons. Data for patients who completed the usual care components (SQiD and CAM) but did not progress to consent for data to be included in the study or did not consent or withdrew consent were not included in the analysis.

## Results

Recruitment occurred between 7th April 2012 – 8th May 2015 at site one, and from the 19th of March 2014 – 2nd of September 2015 at site two. A total of 1175 patients were identified, 509 of whom were assessed for eligibility, with a total of 142 patients consented to be part of the study (Fig. 1).

The median age across all consenting participants was 68 years (IQR 56–77.5 *n* = 122) and of these, 56 (47%) were female (Table 1). Major cancer types were lung 14 (19%), gastric or other upper GI 11 (15%) and breast 10 (14%). A third of the patients had stage IV cancer, and cerebral metastases were present in 9 (7%) cases. In total, 88 (72%) of 122 participants admissions were for symptom management. The SQiD respondent and the patient resided together for 69 (57%) participants. Median length of hospital stay was 12 days (IQR 5–18). Median Australia-modified Karnofsky Performance Status (AKPS) [25] was 70 (IQR 50–70). The median AKPS score for the first centre was 70 (IQR 50–80), and for the second centre was 65 (IQR 60–70) with no significant difference in the distributions between the two centres (Wilcoxon Two-sample test z = − 0.02, *p* = 0.983.

**Table 1:** Demographic characteristics of the study participants

	Primary analysis *N* = 73	Secondary analysis *N* = 122
**Gender**		
Male	33 (42%)	62 (51%)
Female	39 (53%)	56 (46%)
**Admitting team**		
Medical Oncology	47 (64%)	85 (70%)
Radiation Oncology	17 (23%)	22 (18%)
Palliative Care	7 (10%)	9 (7%)
**Admission indication**		
Symptom management	55 (75%)	88 (72%)
**Tumour type**		
Lung	14 (19%)	23 (20%)
Gastric and other upper GI	11 (15%)	15 (12%)
Breast	10 (14%)	14 (11.5%)
Prostate	3 (4%)	13 (11%)
Colon	8 (11%)	9 (7%)
Other	23 (31.5%)	39 (32%)
**Tumour stage**		
Stage I or II	8 (11%)	10 (8%)
Stage III	12 (16%)	19 (15%)
Stage IV	50 (68.5%)	85 (69.7%)
**Site of metastasis**		
No documented metastasis	20 (27%)	32 (26%)
Bone	18 (25%)	24 (20%)
Liver	10 (14%)	13 (11%)
Lung	4 (5.5%)	7 (6%)
Brain	3 (4%)	9 (7%)
Multiple sites	12 (16%)	25 (20.5%)
Unknown/missing	6 (8%)	10 (8%)
**ECOG Score**		
1 or less	22 (30%)	31 (25%)
2	22 (30%)	40 (33%)
3 or more	19 (26%)	34 (28%)
Unknown/missing	10 (14%)	17 (14%)
**SQiD informant cohabitation with patient**	41 (56%)	69 (57%)
	Median (IQR)	Median (IQR)
Age	68 (60.5–78)	68 (56–77.5)
Australia-Modified Karnofsky Score	70 (50–80)	70 (50–70)

*GI* Gastro intestinal, *IQR* Interquartile range

Compared with the source population, study participants were significantly older (median age 68 vs 65, *p*-value 0.006), and had longer length of stay (median 12 vs 5 days, *p*-value< 0.001). (Table 2) There were no differences in gender distribution between participants and non-participant groups. The median time difference between administration of the SQiD and the psychiatrist interview was 26 h (IQR 19–71), and 24 h (IQR 1–23) between the CAM and psychiatrist interview.

**Table 2:** Comparison of characteristics of participants versus background population

	Background population *N* = 2352	Primary analysis n = 73	Secondary analysis *n* = 122
Female (%)	45%	54%^ns^ *p*-value = 0.146	47% ^ns^ *p*-value = 0.676
Age Median (IQR)	65 (54–74)	68 (60.5–78)* *p*-value = 0.010	68 (56–77.5)* *p*-value = 0.006
LOS Median (IQR)	5 (3–10)	12.5 (6–18.5)* *p*-value < 0.001	12 (5–18)* *p*-value < 0.001

^ns^ – non-significant difference between participants and background population

*Statistically significant differences between participants and background population, tested using Mann-Whitney test

*IQR* Interquartile Range, *LOS* Length of stay

The diagnosis of delirium based on psychiatrist interview was made in 27/71 (38%). Motoric subtype of those interviewed was 4/71 hyperactive, 16/71 hypoactive, and 6/71 mixed [21]. The SQiD identified 18 cases of delirium, a third of which were true positives. The primary endpoint of NPV of the SQiD compared to psychiatrist interview was 73% (95% CI 65–79) (Table 3).

**Table 3:**
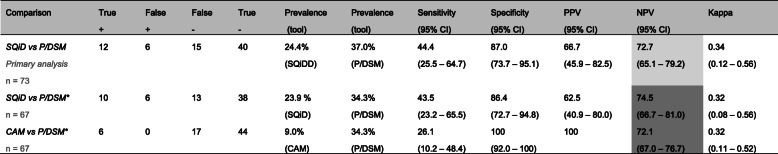
Comparison of delirium test diagnostic values and measures of agreement

*PPV* Positive predictive value, *NPV* Negative predictive value, *SQiD* Single Question in Delirium, *CAM* Short Confusion Assessment Method, *P/DSM* Psychiatrist DSM interview.

Cohen’s kappa statistic (kappa) values above 0.75 denote excellent agreement, 0.40 to 0.75 fair to good agreement, 0.21 to 0.4 as fair and below 0.20 poor agreement.* Restricted to patients who had all three measures recorded, SQiD, CAM and P/DSM *N* = 67.

Primary analysis.

Secondary analysis

Among those patients who completed all tests (*n* = 67), both SQiD and CAM had similar NPV (75% v 72%) and kappa coefficient (0.32), with SQiD exhibiting higher sensitivity (44% vs 26%) but lower specificity (87% vs 100%). Of the 16 patients with hypoactive delirium diagnosed by psychiatrist interview, the SQiD identified five of the cases, while the CAM identified one. Overall, the CAM identified a low number of delirious patients, but all CAM positive patients identified delirium correctly.

The remaining primary and secondary endpoints are presented in Table 3.

## Discussion

Our study demonstrates that the SQiD has good performance and is comparable with the CAM in terms of NPV against psychiatric interview, the diagnostic reference standard. This is an important attribute of the study as without a robust standard applied systematically across a validation study, in keeping with recommended delirium diagnosis methodology [3], the validity of a detection tool cannot be established. Our study found fair correlation of both SQiD and CAM in relation to the reference standard. The CAM had a better PPV but a low sensitivity, whereas the SQiD had better sensitivity. The CAM however, found much lower number of patients with delirium than the reference standard, but with the caveat that all those identified as being delirious or non-delirious by the CAM were correctly identified, hence the contrast between Sensitivity (26%) and Specificity (100%) of the CAM.

Methodological strengths of our study include a clear description of patient characteristics and setting that mitigated case ascertainment bias and the method which prevented training bias due to study flow.

The use of an accurate and reproducible reference standard with a clear description of reference-rater training and characteristics is of central importance. This was a pragmatic, clinically embedded, ‘real world’ study. The SQiD is simple and provides a pragmatic approach to support uptake and also enabled families to be involved in discussions with bedside clinicians regarding delirium. Informal feedback suggests it has good face validity to engage staff in actively seeking delirium. A better understanding of what happens after the SQiD question is asked may benefit future iterations and aid an understanding of the role for the SQiD in staff education and the promotion of helpful discussion between carers and staff.

Current guidelines do not support routine delirium screening in Oncology settings due to lack of evidence [26], they do however provide the following advice “any changes in cognitive or emotional behaviour or psychomotor activity suggestive of delirium are present, a trained healthcare professional with expertise in evaluating delirium should carry out a clinical assessment to confirm the diagnosis of delirium” which lends support to engaging staff and family in detecting this change. While the SQiD does not replace clinical assessment its NPV may support clinicians in identifying patients for further review.

Of the 16 patients with hypoactive delirium, 6 were identified on SQiD, the CAM identified only one. The detection of hypoactive delirium is an important consideration in delirium detection tools as typically hypoactive delirium poses more difficulties for clinical identification. Given the small numbers in each motoric sub-group, this observation needs to be interpreted with caution, however this is one aspect of the SQiD which may support further investigation.

As the SQiD is not directly asked of the patient, it is not important that patients speak English. This may even be an advantage but at this point it is a theoretical advantage not tested by this study which was constrained by exclusion of patients with insufficient English fluency for consent and psychiatrist interview.

Limitations of our study relate to a non-consecutive sample and exclusion of some patient groups. The study population had some statistically-significant differences to the source population – they were older and had longer length of stay. Further it is of note that delirium detection rates in our sample are not presented as representative of the base population and cannot therefore be used to estimate delirium incidence or prevalence. The rates detected in the study population were however not dissimilar to incidence or prevalence established in several other medical in-patient cohorts [13, 14, 24, 27–29]. A further limitation was that SQiD administration appeared to be dependent on prompting by senior staff, making recruitment subject to variation due to competing demands and staffing. The time between SQiD, CAM and psychiatric interview was also generally longer than anticipated in the protocol. This can have implications for diagnosis as delirium symptoms fluctuate over time. There is a possibility of recruitment centre bias however in terms of Australia-modified Performance Status (AKPS) [25] no bias was detected. Lastly, the study did not reach the planned sample size, which resulted in lower power and wider confidence intervals.

Although CAM training for study staff was conducted it fell short of that recommended for diagnostic purposes and we did not achieve the sensitivity and specificity in using the CAM that has been reported elsewhere [9]. One of the reasons the CAM was included as a comparator to the SQiD was to determine if it was an option for routine use in our clinical setting. With the training possible in the cancer clinical context this was not the case, and we note that other recent studies have reported similar short-comings in training staff so that optimum sensitivity and specificity of the CAM has not been achieved in those clinical and clinical research settings [12, 13].

A further limitation is that the only information gathered about the SQiD informant was co-habitation with the patient. Cognitive testing to understand how family and carers interpret the SQiD question, the subsequent discussion, and understanding of the meaning of question prior to the study may be of relevance, and is recommended for consideration in future studies.

Finally, a number of patients in our study were excluded due to lack of English fluency (*n* = 43). Therefore, conclusions about the utility of the SQiD in patients across cultural and linguistic backgrounds other than that of English speakers remains untested. Similarly, the SQiD may be of lower utility for patients who live alone or have limited contact with other people. Nevertheless, of 142 patients in our study, only five were excluded due to not having a person available to answer the SQiD.

### Implications for training and practice

The SQiD may engage nurses, medical staff, carers and/or patients in discussion regarding the components of syndromic delirium. The use of the SQiD by clinical staff may reinforce the concept that delirium is an important medical issue for staff, patients and family/carers.

### Implications for research

Other studies have compared short tests of cognition or attention [4, 8, 12, 30, 31] however, to our knowledge, having a clinician directly approach the main carer with a conversational approach and a simple question regarding recent changes in cognition, although it makes common sense/good clinical practice, has not been previously evaluated. Future research questions may focus on the content of the discussion that ensues following administration of the SQiD, and the ability of the tool to engage staff and family/carers in looking for changes that would indicate a need for further clinical delirium assessment.

The combination of a detection tool with high sensitivity followed by one with high specificity may be a useful approach to screening and could be addressed in future studies. Since inception of this study other short and brief tools have appeared in the peer review literature, including the 4AT [30] and more recently the delirium RADAR [31]. These tools are promising either alone or in combination, however they may not have the same qualities of engaging carers and staff that is possible with the SQiD, which again raises hypotheses relating to combined administration of short tests.

## Conclusion

The SQiD had NPV comparable to the CAM when used with a pragmatic level of training in our clinical setting. The SQiD has favourable psychometric properties; on face value it is feasible and pragmatic for staff, acceptable to carers and patients and engages staff, family and carers in a shared and educational goal. The SQiD can contribute to delirium detection and clinical care for hospitalised cancer patients.

## Supplementary information

Additional file 1. SQID QUESTIONNAIRE. (PDF 115 kb)

## Data Availability

From corresponding author, on request.

## Abbreviations

4AT: 4 A’s Test
CAM: Confusion assessment method (used throughout to signify short or brief confusion assessment method)
MDAS: Memorial delirium assessment scale
MMSE: Mini-mental state examination
Nu-DESC: Nursing delirium screening scale
RADAR: Recognizing acute delirium as part of your routine
SQiD: Single question in delirium
AKPS: Australia-modified karnofsky performance status
DSM: Diagnostic and statistics manual of mental disorders
DSM IV R: Diagnostic and statistics manual of mental disorders fourth edition (revised)
DSM 5: Diagnostic and statistics manual of mental disorders fifth edition
ICD-10: International classification of diseases, 10th revision
LNR: Human research and ethics committee low negligible risk
NPV: Negative predictive value
PPV: Positive predictive value
P/DSM: Psychiatrist DSM interview
SAS: Statistical analysis system
