# Supplementary material for: “SQiD, the Single Question in Delirium; can a single question help clinicians to detect delirium in hospitalised cancer patients?” running heading Single Question in Delirium” (Bcan-D-20-01665)
Source: BMC Cancer. 2021 Jan 18;21:75. doi: 10.1186/s12885-020-07504-x (PMC7814717; doi:10.1186/s12885-020-07504-x)
Supplement: Supplementary file 1 — SQID QUESTIONNAIRE. (PDF 115 kb) [file 12885_2020_7504_MOESM1_ESM.pdf]

The Prince of Wales Cancer Centre

University of New South Wales
The Prince of Wales Hospital
Barker Street Randwick NSW 2031
Sydney Australia

Principal Investigator: Dr Meg Sands

Department Of Palliative Care
Tel: (+61 2) 9382 4020
Fax: (+61 2) 9382 1579

SQiD

– Testing the performance of a Single Question in Delirium case finding.

SQID QUESTIONNAIRE

Name:___________________________

1. Date:_________________________________

2. Time:____________________________________

3. **ASK FAMILY/FRIEND:**

"Do you feel that [patient's name] *has been more confused lately?"*

RECORD RESPONSE (tick):

- YES _____________
- NO ________________

4. **RELATIONSHIP TO PATIENT (eg Mother, Father, Sister, Son, Daughter, etc):**

____

5. **DOES THIS PERSON LIVE WITH THE PATIENT?**

- YES_____________
- NO_____________

TIME_______________

NAME/SIGN____DATE_______________

Version 5 April 19th 2012
A Centre for Multidisciplinary Cancer Treatment and Research
A Facility of South Eastern Sydney Local Health District
